# Detection of Rabies Antigen in the Saliva and Brains of Apparently Healthy Dogs Slaughtered for Human Consumption and Its Public Health Implications in Abia State, Nigeria

**DOI:** 10.1155/2013/468043

**Published:** 2013-12-12

**Authors:** P. P. Mshelbwala, A. B. Ogunkoya, B. V. Maikai

**Affiliations:** ^1^Department of Veterinary Medicine, Ahmadu Bello University, Zaria, Nigeria; ^2^Department of Veterinary Public Health and Preventive Medicine, Ahmadu Bello University, Zaria, Nigeria

## Abstract

The study was carried out in eight dogs slaughtering outlets within four Local Government Areas of the State for the determination of rabies antigen in the saliva and brain of apparently healthy dogs slaughtered for human consumption. A total of one hundred (100) samples each of saliva and brain were collected before and after slaughter, respectively, between April to June, 2013, in the selected areas. The saliva was subjected to rapid immune-chromatographic test (RICT) while direct fluorescent antibody test (DFAT) was carried out on the brain samples. Structured questionnaire was administered to nineteen (19) dog meat processors comprising 18 males and 1 female in the selected areas. Sixty four percent of the samples tested were from female dogs while 36% were from males, 5% tested positive for rabies antigen with the use of both tests; there was no statistical association between sex and rabies status of the dogs sampled (*P* > 0.05). Butchers bitten during the course of slaughtering were 94.7% out of which 72.8% utilized traditional method of treatment and only 27.8% reported to the hospital for proper medical attention. This study has established the presence of rabies antigen in apparently healthy dogs in the study area.

## 1. Introduction

Rabies, though a vaccine preventable disease, still accounts for the death of over 50,000 people worldwide; 95% of these fatalities were apportioned to Africa and Asia [1] while 98% of the cases were caused by rabies infected dog bite [2]. Worldwide, lines of evidence abound supporting the fact that dogs shed rabies virus in their saliva (Nottidge, 1994) [3]. Fekadu et al. [4] have established that rabies infected dogs that recovered shed rabies virus in their saliva intermittently, and this shedding continued for about 7 months while the dog remained apparently healthy; this represents a carrier status which was previously described in Nigeria [5]. Inapparent infection and recovery from clinical disease with resultant persistent or intermittent shedding of rabies virus have affected the overall effort in rabies eradication and control in most parts of the world [6–8]. The most dangerous aspect of carrier dogs is that bites from such dogs are usually not recognized as an exposure that will stimulate appropriate postexposure treatment. Consequently human deaths occur from exposure buried in an erratic incubation period where dogs show no sign suggestive of rabies [9].

Abia State shares border with Cross River State. Recently, 8 people died and the cause was traced to dog bite [10]. Those dogs were disposed off before reports got to Veterinary authorities, and it has been suggested that those suspected dogs must have been consumed before the onset of clinical signs thereby precluding confirmatory diagnosis [10].

Work on rabies in apparently healthy dogs has been carried out in different parts of the country [7, 11–13]. However, information on rabies in Abia State is wanting despite rabies outbreak in neighbouring states. There still exists an eclipse of information on rabies epidemiology in Abia state; there is therefore a need to find out the status of rabies in the state, especially carrier status. The study was undertaken to find out the presence of rabies antigen in the saliva and brain of apparently healthy dogs slaughtered for human consumption in Abia State and to find out the practices of dog meat slaughtering by butchers who fall within the high risk group.

## 2. Materials and Methods

### 2.1. Study Area

Located in the south-eastern part of Nigeria, Abia State lies within approximately latitudes 4°40′ and 6°14′ north of the equator and longitudes 7°10′ and 8° east of the Greenwich meridian. The state shares common boundaries to the north with Ebonyi State; to the south and southwest with Rivers State; and to the east and southeast with Cross River and Akwa lbom States, respectively. To the west is Imo State and to the northwest is Anambra State. The state covers an area of about 5,243.7 sq·km which is approximately 5.8 percent of the total land area of Nigeria. The study was carried out in Umuahia north, Ekwuano, Osisioma, and Isianlangwa north Local Government Areas of the State.

### 2.2. Sampling Method

Convenient random sampling as described by Mike [14] was employed. The most accessible units of the population were used. Live dogs bought or brought for slaughter were sampled in this designated Local Government Areas.

### 2.3. Use of Questionnaire

Structured questionnaire was administered to dog meat processors in the selected areas to obtain information on frequency of bite and method of treatment following bite.

### 2.4. Processing of Samples

#### 2.4.1. Saliva

Saliva of dogs presented for slaughter was sampled using the sterile swab stick which was inserted into the mouth of the dog before slaughter. The swab was then inserted into an assay buffer tube and stirred to ensure a good sample extraction. The immune chromatographic test cassette was removed and placed horizontally. Using a sterile dropper, three drops of the extracted sample were dipped into the sample hole in the cassette, and the result was interpreted within 5–10 minutes according to manufacturer's instruction (BioNote). One hundred saliva samples were swabbed.

#### 2.4.2. Brain

Brains of dogs, whose saliva was previously tested, were extracted using the method described by Kaplan and Koprowski [15]. It was then placed in polythene bag and transported to National Veterinary Research Laboratory, Vom Plateau State, and stored in a deep freezer for fluorescent antibody test. All stored samples were removed from the deep freezer and were allowed to thaw and smears were made on slides immediately after opening the brain and then subjected to fluorescent antibody test (FAT) as described by Dean et al. [16].

### 2.5. Data Analysis

In this study, data generated were analyzed using the Statistical Package for Social science (SPSS) Version 17.0 to carryout descriptive analysis. Chi-square was used to test for association between categorical variables. Values of *P* < 0.05 were considered significant.

## 3. Result

**Figure 1:**
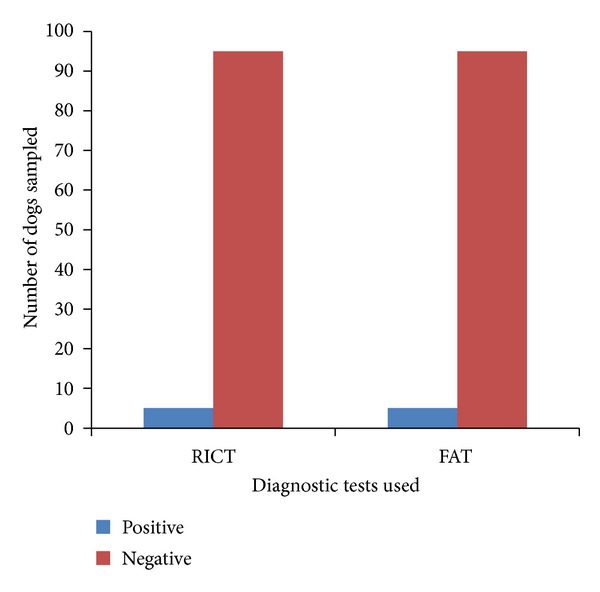
The results of the tests used for saliva and brain samples.

**Figure 2:**
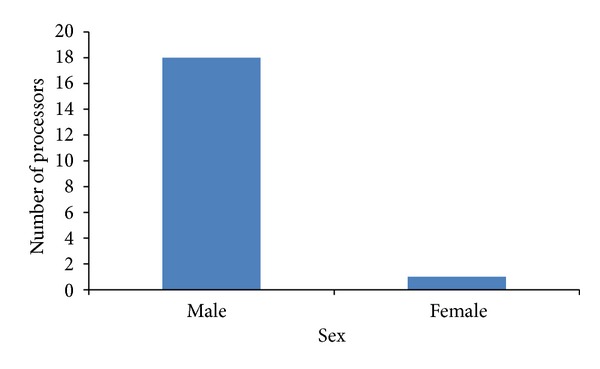
Sex distribution of processors of dog meat in Abia state.

**Figure 3:**
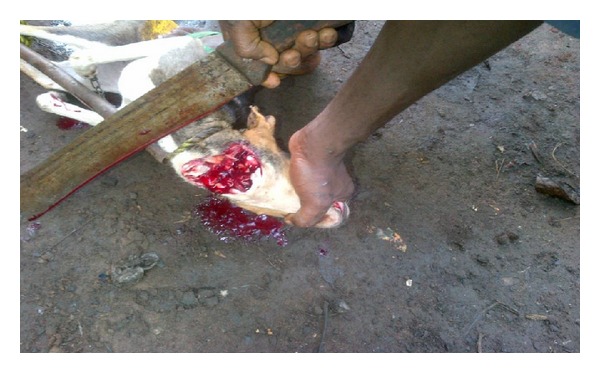
Butcher slaughtering dog for meat, using his bare hands to muzzle the mouth.

**Figure 4:**
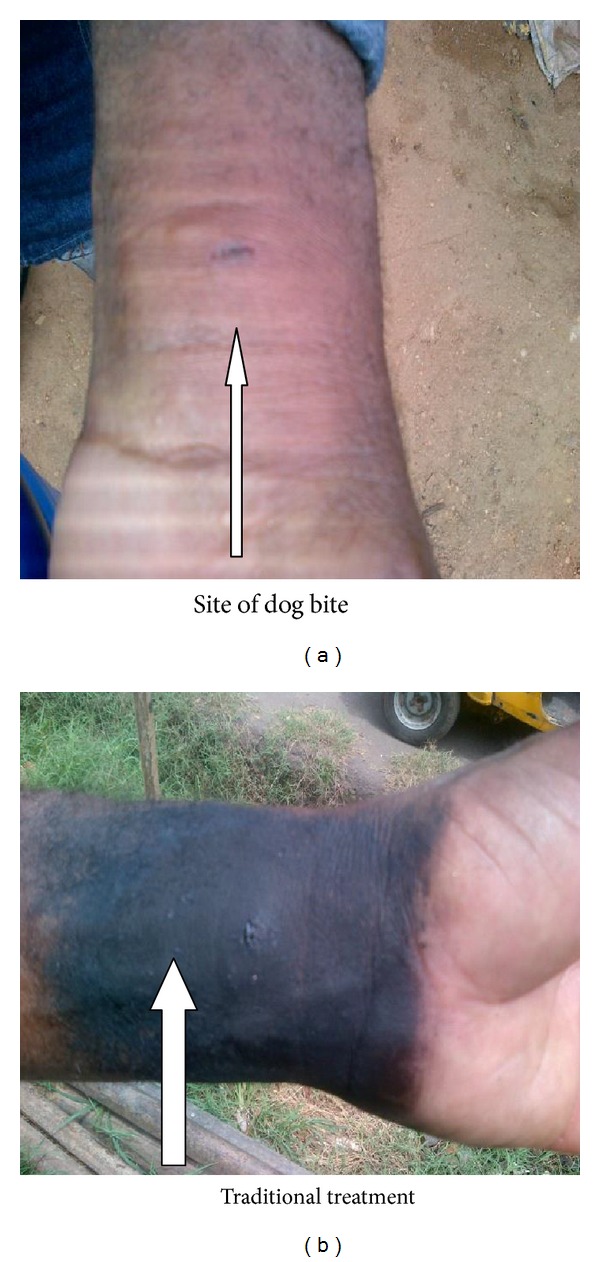
Site of dog bite sustained by dog meat processor in Abia state, Nigeria, and traditional treatment.

This study showed that out of the 100 dogs sampled more females (64%) than males 36% were slaughtered (Table 1). All the positive cases (5%) were females and also were indigenous breeds (Table 2). There is a total agreement between the results obtained for DFAT and RICT for all the samples analysed in this study (Figure 1). None of the dogs meat processors were previously vaccinated against rabies; although the majority (94.7%) were previously exposed to dog bite during the slaughtering process, only 5.3% were not exposed to dog bite (Table 3). On exposure to bite, 72.7% sought for traditional method of treatment while 27.8% reported to the hospital (Table 4); there was no significant association between sex of dogs slaughtered and positivity (0.08). Butchering was seen as occupation of male, although one of them happens to be a woman (Figure 2); there was no association between sex and butchering. The butcher's educational profile revealed the following: 68.4% had primary education, 26.6% did not have any formal education, and 5.3% had tertiary education. Butchers handle dogs in a manner that exposed them to dog bite (Figure 3); on exposure to dog bite, butchers utilized traditional method of treatment, using traditional leaves and teethes of the offending dog, which is burnt to aches, mixed with native gin; part is given to the victim to drink and vomit while the rest is applied at the site of bite (Figure 4).

## 4. Discussion

The 5% prevalence of rabies antigen in the saliva and brains of apparently healthy dogs sold for human consumption in Abia State as demonstrated in this study is also in agreement with that of earlier work. [12] which established that apparently healthy dogs excrete rabies virus in their saliva for long period of time without showing clinical signs. Aghomo and Rupprecht [17] also reported lower prevalence in South Western Nigeria. In the study carried out in Ethiopia (Fekadu, 1975) dog inoculated with rabies virus recovered from clinical signs and continued to excrete rabies virus in its saliva. The result of this finding further suggests possible existence of carrier state among apparently healthy dogs in Abia State, Nigeria.

None of the butchers in this study were previously vaccinated against rabies, although majority of them (94.7%) were previously bitten during the course of slaughtering which is in agreement with the findings of Garba et al. [18] in Northern Nigeria. Following dog bite, majority of the butchers sought traditional method of treatment, as against proper medical care, a practice which will further complicate the problem as none of the methods used have been tested to be effective. This is in agreement with the report of Ogunkoya [19]. In his report, he opined that traditional methods of treatment could militate against the control and eradication of rabies. The low educational level of the butchers could be the reason why they engaged in such practices.

Butchers in the study area handled dogs without taking adequate precaution which is the main reason why most of them sustained dog bite in the course of slaughtering; some even muzzled the dogs mouth using their bare hands, a practice which is capable of exposing them to bite or salivary contamination. This finding is similar to that of Garba et al. [18], Northern Nigeria. Rabies infection through skinning and handling of animals has been reported [20]. In our study, it can be seen that the manner in which butchers handled dogs before slaughter calls for concern, as this may further increase the likelihood of exposure to rabies.

The age, sex, breed, and location of dogs slaughtered during the course of the study were also considered. In this study, more females were slaughtered than males; this finding differs from the findings of Aliyu et al [12] and Ajayi et al. [7] in Northern Nigeria. The reason for attributing more females may be due to the practice of using male dogs for burial ceremony in some parts of the state, which makes the prices of male dogs go higher than females and also scarce. There was no association between sex and rabies status of the dogs sampled (*P* > 0.05). All the dogs sampled were sourced from their neighbourhood; this finding is in agreement with that of Garba et al. [18]; this is an indication that these dogs actually lived a normal life and continued to excrete the virus in their saliva without showing clinical signs. This form of rabies is the dangerous type as people are only aware of the violent form of rabies [19]. Human deaths from bites by owned dogs have been reported in Nigeria [21].

Dog butchering is seen as the business of male, but in this study, one of the butchers was a female and was pregnant at the time of sample collection. This finding differs from that of Garba et al. [18]; in their work males were seen as butchers. In northern Nigeria, females do not engage in some businesses based on religious restriction, especially Muslims; however in our studies in the eastern part of Nigeria, female movement is not restricted and there are no specifications of the type of business females are expected to engage in. Majority of the butchers had only primary education as only one had tertiary education; this could be the reason why they handled dogs without taking precaution. There is a total agreement between the results of all the tests used for rabies as all gave 5% prevalence. In conclusion, this study has established the presence of rabies antigen in the saliva and brains of apparently healthy dogs slaughtered for human consumption in Abia State signifying its endemicity as it is in other states where studies have been conducted. This study also revealed the practices of dog slaughtering as a possible source of exposure to dog meat processors in Abia State. Hence mass enlightenment program for dog meat processors, consumers, and children in the state is highly recommended.

## Figures and Tables

**Table 1:** Distribution of dogs samples according to sex and positivity in Abia state, Nigeria.

Sex	Number tested	Number positive (%)	
Male	36	0	0
Female	64	5	7.8

Total	100	5	5

**Table 2:** Breed and positivity distribution.

Breed	Number tested	Number positive (%)	
Endogenous	96	5	5.2
Mixed	3	0	0
Exotic	1	0	0

Total	100	5	5%

**Table 3:** Dog meat processors previously bitten during slaughtering processes.

	Number of butchers	Percentage (%)
Once bitten	18	94.7
Never Bitten	1	5.3

Total	19	100

**Table 4:** Method of treatment for victims of dog bite.

Method of treatment	Number of processors	Percentage
Hospital	5	27.8
Traditional	13	72.8

Total	18	100
